# Conjugated nitrosoalkenes as Michael acceptors in carbon–carbon bond forming reactions: a review and perspective

**DOI:** 10.3762/bjoc.13.220

**Published:** 2017-10-23

**Authors:** Yaroslav Dmitrievich Boyko, Valentin Sergeevich Dorokhov, Alexey Yu Sukhorukov, Sema Leibovich Ioffe

**Affiliations:** 1 N. D. Zelinsky Institute of Organic Chemistry, 119991, Leninsky prospect, 47, Moscow, Russia; 2 Higher Chemical College, D. Mendeleev University of Chemical Technology of Russia, 125047, Miusskaya sq., 9, Moscow, Russia

**Keywords:** carbon–carbon bond formation, Michael addition, nitrosoalkenes, oximes, total synthesis

## Abstract

Despite of their chemical instability and high reactivity, conjugated nitrosoalkenes are useful intermediates in target-oriented organic synthesis. The present review deals with carbon–carbon bond forming reactions involving Michael addition to α-nitrosoalkenes with a particular focus on recent developments in this methodology and its use in total synthesis.

## Introduction

Conjugated nitrosoalkenes (NSA) are close analogs of α-nitroalkenes, which are important Michael acceptors in organic synthesis [1–5]. Unlike the parent α-nitroalkenes, α-nitrosoalkenes are unstable species usually generated in situ, and their synthetic potential has been studied in significantly less detail [6–7]. Because of relatively short lifetimes of α-nitrosoalkenes, conjugate addition of nucleophiles is complicated by side reactions, such as polymerization. Successful coupling of nitrosoalkenes with nucleophiles is highly challenging and depends on many factors such as temperature, concentration, solvent and, especially, the nature of the α-nitrosoalkene precursor. Nevertheless, Michael addition of C-nucleophiles to α-nitrosoalkenes opens access to synthetically valuable α-branched oximes, which can be further utilized as useful intermediates in total synthesis. The high potential of this carbon–carbon bond-forming strategy has been recognized since 1970s in works of Corey, Oppolzer, Gilchrist and others. However, at that time, researchers faced substantial problems associated with low chemoselectivity of reactions with nitrosoalkenes generated in situ from the corresponding α-halooxime precursors as well as with the need of a large access of the nucleophile.

In the recent years, there has been a considerable growth of interest to the conjugate addition of carbon-centered nucleophiles to α-nitrosoalkenes. This is mainly due to the development of new precursors, from which nitrosoalkenes are generated at low stationary concentrations under very mild conditions that allows to suppress side reactions and to use a stoichiometric amount of nucleophile. Under these conditions, Michael addition reactions with nitrosoalkenes can be realized in an intramolecular fashion that opens access to complex bridged and fused carbocyclic frameworks bearing several contiguous stereogenic centers.

In this review, we attempted to summarize up-to-date data on the successful coupling of α-nitrosoalkenes with C-nucleophiles (classified according to a nucleophile type) emphasizing on examples from target-oriented synthesis as well as to analyze growing points in this methodology. The review does not deal with cycloaddition reactions of nitrosoalkenes. For this aspect of nitrosoalkene chemistry, the reader can be referred to earlier general reviews [6–10].

## Review

### Precursors of conjugated nitrosoalkenes

**Scheme 1:**
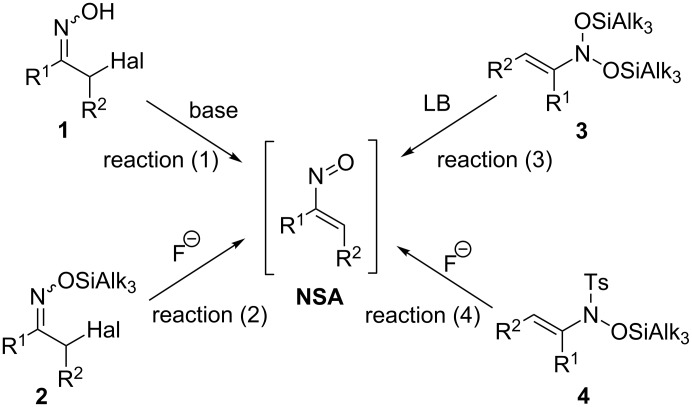
Precursors of nitrosoalkenes **NSA**.

The way how α-nitrosoalkenes are generated is essential for achieving a successful coupling with a particular nucleophile and for prevention of side reactions. There are several sources of α-nitrosoalkenes as shown in Scheme 1. Conventional precursors of α-nitrosoalkenes are α-halooximes **1** (or halo-substituted nitroso compounds [11–12]), which undergo deprotonation/halide elimination upon treatment with a base [13] (Scheme 1, reaction (1)). A disadvantage of this method is that **NSA** are generated fast and high stationary concentrations are achieved facilitating polymerization. Furthermore, an excess of nucleophile is needed since it also serves as a base. A milder method was suggested by Denmark, who used α-halooximes silyl ehers **2** as precursors of conjugated nitrosoalkenes upon treatment with TBAF at low temperatures (Scheme 1, reaction (2)) [14–16]. Importantly, the rate of nitrosoalkene formation can be controlled by the bulkiness of the silyl group. Another way of nitrosoalkene generation employs their stable nitrosoacetals of type **3** (*N*,*N*-bis(silyloxy)enamines) [17–18]. The latter eliminate (Alk_3_Si)_2_O upon the action of Lewis bases generating the corresponding nitrosoalkenes at low stationary concentrations in a controllable manner. Furthermore, synthetic precursors of nitrosoacetals **3** are not halocarbonyl compounds as in the case of oximes **1** and **2**, but aliphatic nitro derivatives, which are readily transformed into enamines **3** by double silylation involving an internal redox process [19–21]. This allows the preparation of nitrosoalkenes, which are difficult to access by the first two routes. Tanimoto et al. [22] recently suggested *N*-siloxysulfonamides **4** as stable sources of unsaturated nitroso compounds (Scheme 1, reaction (4)). However, this approach was used only for the generation of nitrosoallenes so far.

### Addition of C–H acids anions to conjugated nitrosoalkenes

**Scheme 2:**
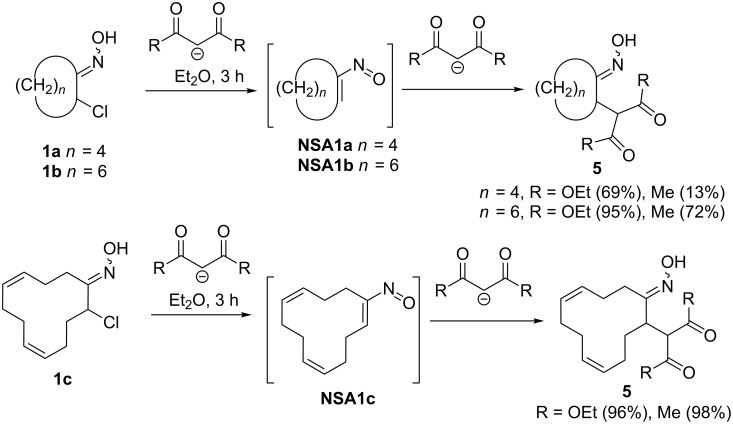
Reactions of cyclic α-chlorooximes **1** with 1,3-dicarbonyl compounds.

Stabilized enolates and their equivalents are the most studied C-nucleophiles in Michael addition reactions with nitrosoalkenes. A first systematic study in this area was done by Ohno and co-authors [13,23], who reported the addition of diethyl malonate and acetylacetone anions to cyclic nitrosoalkenes **NSA1a–c** generated from the corresponding α-chloroketones (2-chlorocyclohexanone oxime (**1a**), 2-chlorocyclooctanone oxime (**1b**) and 12-chloro-сyclododeca-4,8-dien-1-one oxime (**1c**)) upon the action of an excess of nucleophile (Scheme 2). It was found that oximes **1b** and **1c** react both with diethyl malonate and acetylacetone, providing adducts **5** in high to quantitative yields. Six-membered cyclic oxime **1a** afforded the corresponding products only in moderate yields owing to the formation of undesired side products and polymerization of the α,β-unsaturated nitroso compound. This early example demonstrates the high dependency of Michael addition to nitrosoalkenes on the substrate structure. However, the need of an excess of nucleophile significantly limits the application of this protocol.

**Scheme 3:**
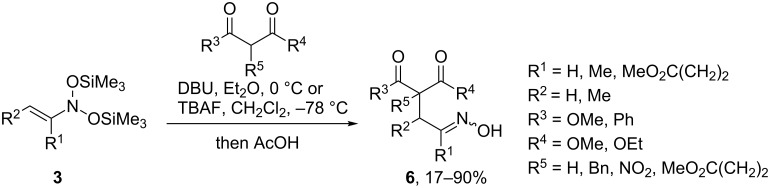
C-C-coupling of *N*,*N*-bis(silyloxy)enamines **3** with 1,3-dicarbonyl compounds.

The development of improved methods for the generation of α-nitrosoalkenes have led to more extensive studies of their reactions with enolates. Thus, our group has demonstrated that nitrosoacetals of nitrosoalkenes (*N*,*N*-bis(silyloxy)enamines **3**) smoothly react with 1,3-dicarbonyl compounds in the presence of TBAF or DBU to give the corresponding oximes **6** in moderate to good yields (Scheme 3) [24]. The process is likely to involve a nitrosoalkene intermediate, which is generated upon the action of TBAF or the anion of the 1,3-dicarbonyl compound on *N*,*N*-bis(silyloxy)enamine **3**. Unlike malonic acid derivatives and β-keto esters, 1,3-diketones produced only complex mixtures of products in the reaction with *N*,*N*-bis(silyloxy)enamines **3** under these conditions.

**Scheme 4:**
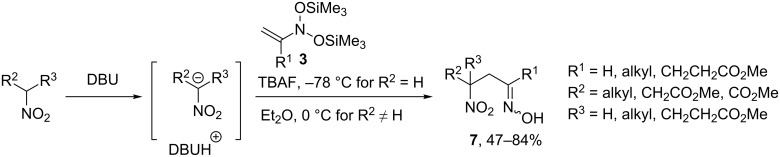
Reaction of *N*,*N*-bis(silyloxy)enamines **3** with nitronate anions.

Nitronate anions also react as C-nucleophiles with *N*,*N*-bis(silyloxy)enamines **3** producing β-nitrooximes **7** in good yields (Scheme 4) [20,25]. Efficient coupling of DBU-nitronate salts derived from primary nitro compounds with enamines **3** was achieved at –78 °C upon the action of TBAF. However, for less reactive secondary nitronates reactions were carried out at 0 °C without TBAF in order to keep the concentration of reactive nitrosoalkenes lower.

**Scheme 5:**
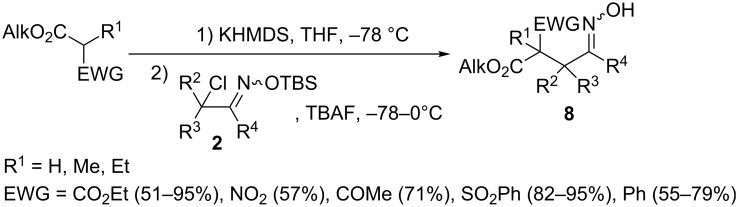
Reaction of α-chlorooximes TBS ethers **2** with ester enolates.

In 2010, Weinreb and co-workers developed a general protocol for coupling α-nitrosoalkenes with carboxylic acid esters bearing electron withdrawing substituents (CO_2_Et, NO_2_, COCH_3_, SO_2_Ph) at the α-position (Scheme 5) [26]. Nitrosoalkenes were generated according to Denmark’s procedure [14] upon the action of TBAF on TBS ethers of α-chlorooximes **2**. Importantly, TBS oxime ethers of various halo-substituted aldehydes and ketones including cyclic ones were successfully used as nitrosoalkenes precursors to give oximes **8** in good to high yields.

**Scheme 6:**
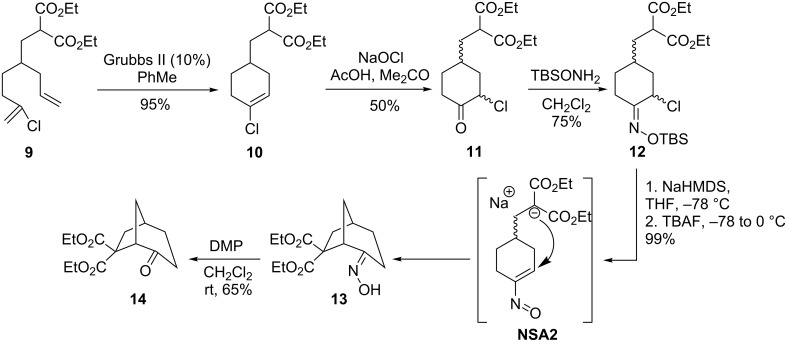
Assembly of bicyclooctanone **14** via an intramolecular cyclization of nitrosoalkene **NSA2**.

The same authors used the advantage of intramolecular Michael addition of enolates to nitrosoalkenes to construct bridged and fused carbocyclic systems [27]. For example, the proposed strategy of bicyclo[3.2.1]octanone **14** assembly is based on a combination of ring closing metathesis reaction and intramolecular addition of the malonate anion to a nitrosoalkene unit (Scheme 6). On the first stage, readily available chlorodiene **9** was subjected to a metathesis reaction with the second-generation Grubbs’ catalyst. The resulting chlorocyclohexene derivative **10** was oxidized to α-chloroketone **11**, which was subsequently transformed into α-chlorooxime TBS ether **12** by standard oximation reaction. The required nitrosoalkene intermediate was generated from α-halo-O-silyloxime **12** upon the action of fluoride anion at −78 °C. It is worth paying attention that the malonate anion is generated prior the addition of TBAF, because of a high instability of the intermediate nitroso compound **NSA2**. Using this procedure, bicyclic oxime **13** was prepared in quantitative yield from precursor **12**. Modification of this strategy allows accessing various bicyclic systems comprising of five-, six- and seven-membered carbo- and heterocycles.

**Scheme 7:**
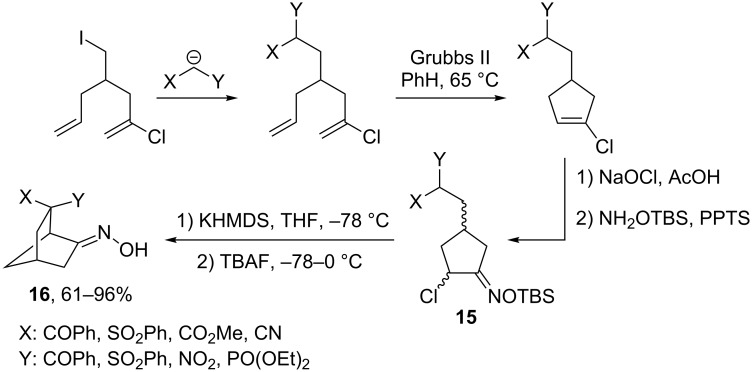
A general strategy for the assembly of bicyclo[2.2.1]heptanes via an intramolecular cyclization of α-chlorooxime TBS ethers **15**.

Not only malonates, but also other CH-acidic fragments such as dicyanomethane and sulfonyl-, nitro- and phosphorylacetic acid residues can be successfully used in the strategy shown in Scheme 6 [28]. This general synthetic scheme opens access to a variety of substituted bicyclo[2.2.1]heptanes **16** via cyclization of α-chlorooxime TBS ethers **15** as shown in Scheme 7.

**Scheme 8:**
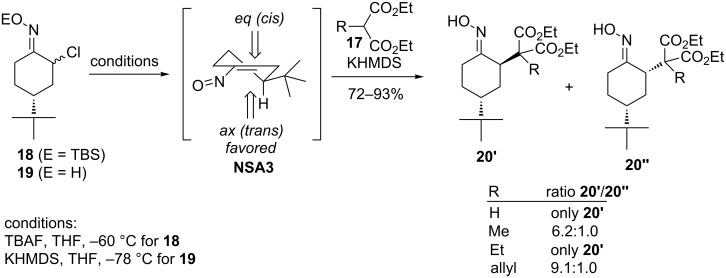
Stereochemistry of Michael addition to cyclic nitrosoalkene **NSA3**.

The stereoselectivity of Michael additions to conjugated nitrosoalkenes is an important issue considering the potential use of this strategy in the synthesis of natural products. Weinreb and co-workers performed a comprehensive investigation of the stereoselectivity of the conjugate addition to α-nitrosoalkenes using both cyclic [29] and acyclic substrates [30]. They have demonstrated that the nucleophilic addition of C-substituted malonic esters **17** to 4-*tert*-butylnitrosocyclohexene **NSA3** (generated either from TBS ether **18** or free oxime **19**) proceeds with high stereoselectivity and affords mainly or exclusively the *trans*-isomers of adducts **20** (Scheme 8) [29]. Expectedly, the stereoselectivity of the Michael addition was not influenced by the method of nitrosoalkene generation as well as by the relative configuration of the α-chlorooxime. The observed stereochemical result can be attributed to an axial attack of the nucleophile on the nitrosoalkene **NSA3**.

**Scheme 9:**
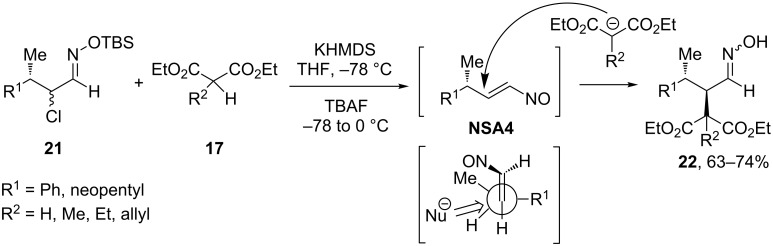
Stereochemistry of Michael addition to acyclic nitrosoalkenes **NSA4**.

More interestingly, the addition of malonic esters **17** to acyclic nitrosoalkenes **NSA4** (generated from oxime TBS ethers **21**) resulted exclusively in *anti*-adducts **22** (Scheme 9) [30]. This stereochemistry is in agreement with the Felkin–Anh model with the most sterically demanding substituent R^1^ lying in perpendicular plane with respect to the C=C double bond. This pattern is general both for nitrosoalkene **NSA4** with phenyl substituent in γ-position (R^1^ = Ph, Scheme 9) and for the compound with a more sterically demanding neopentyl substituent (R^1^ = neopentyl, Scheme 9).

**Scheme 10:**
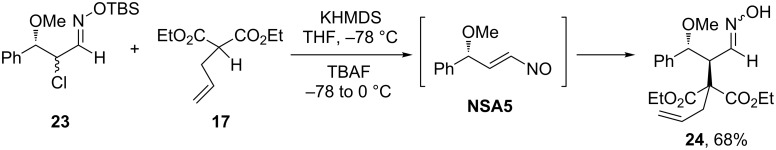
Stereochemistry of Michael addition to γ-alkoxy nitrosoalkene **NSA5**.

Similar to nitrosolakenes **NSA4**, γ-methoxy-substituted nitrosoalkene **NSA5** (generated from TBS-oxime ether **23**) also gave *anti*-isomer of adduct **24** (Scheme 10).

These model studies demonstrate the high stereoselectivity of the Michael addition to both cyclic and acyclic conjugated nitrosoalkenes possessing asymmetric centers. Yet, the stereochemical induction from a chiral nucleophile as well as asymmetric catalysis still remains to be studied in these reactions.

**Scheme 11:**
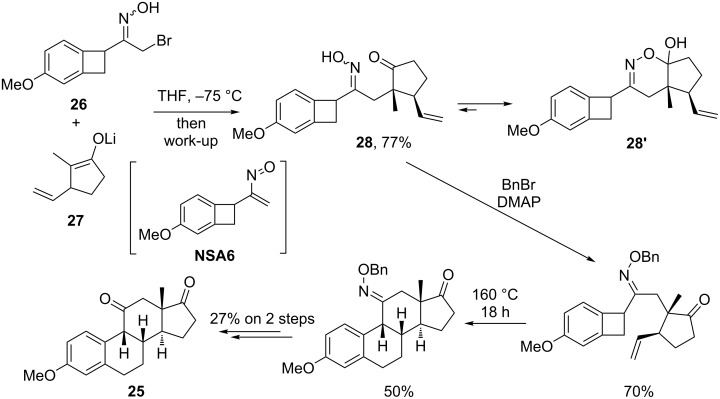
Oppolzer’s total synthesis of 3-methoxy-9β-estra(1,3,5(10))trien(11,17)dione (**25**).

The high efficiency and stereoselectivity of the enolate addition to nitrosoalkenes make this strategy perspective for application in total synthesis that has been recognized since 1970s. Thus, Oppolzer and co-workers exploited the Michael addition of enolates to nitrosoalkenes in their studies towards syntheses of 3-methoxy-9β-estra(1,3,5(10))trien(11,17)dione (**25**) (1977) [31] and (+/−)-isocomene (1981) [32]. The key carbon–carbon bond formation step in the synthesis of steroid **25** was the stereoselective reaction of α-bromooxime **26** with lithium enolate **27** to give oxime **28** via the Michael addition to transient nitrosoalkene **NSA6** [31]. Oxime adduct **28** existed predominantly in a cyclic 1,2-oxazine form (Scheme 11). Subsequent benzylation of oxime **28** and the thermal retro-[2 + 2]/[4 + 2]-cycloaddition cascade followed by hydrolysis of the oximino group gave target steroid **25** with the desired configuration of all stereogenic centers.

**Scheme 12:**
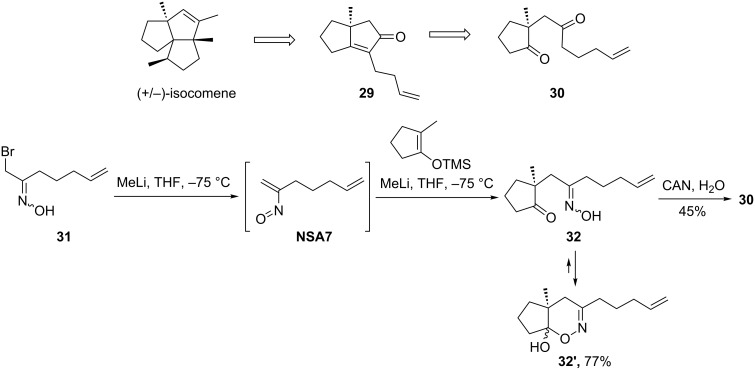
Oppolzer’s total synthesis of (+/−)-isocomene.

In the attempted route to (+/−)-isocomene, Oppolzer and co-workers suggested pentalenone **29**, accessible by an intramolecular aldol condensation/elimination of diketone **30**, as a key precursor. Diketone **30** was prepared in a straightforward manner by C-C-coupling of nitrosoalkene **NSA7** (generated in situ from the corresponding α-bromooxime **31**) with silyl enolate of 2-methylcyclopentanone, and subsequent deoxygenation of the resulting oxime **32** with CAN. Unfortunately, attempts to convert pentalenone **29** into isocomene failed (Scheme 12) [32].

**Figure 1:**
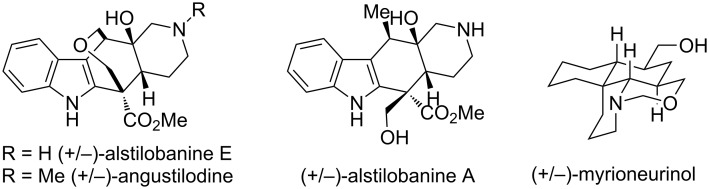
Alkaloids synthesized using stereoselective Michael addition to conjugated nitrosoalkenes.

Recently, Weinreb’s group used the advantage of nitrosoalkenes as Michael acceptors to accomplish total syntheses of several alkaloids, such as (+/−)-alstilobanines A and E, (+/−)-angustilodine [33] and (+/−)-myrioneurinol [34–35] (Figure 1).

**Scheme 13:**
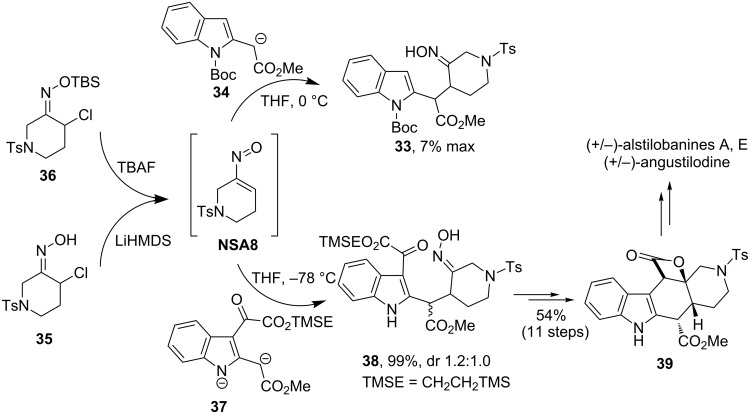
Weinreb’s total synthesis of alstilobanines A, E and angustilodine.

In the total syntheses of alstilobanines A, E and angustilodine, functionalized indole **33** was initially chosen as a key intermediate [33]. However, attempts of its preparation by Michael addition of substituted indole enolate **34** to nitrosoalkene **NSA8**, generated in situ either from the corresponding chlorooxime **35** or its TBS ether **36** failed (Scheme 13). The problem was elegantly solved by using indole dianion **37** as both a nucleophile and a base. Reaction of **37** with α-chlorooxime **35** required only one equivalent of nucleophile and produced adduct **38** in 99% yield. Oxime **38** was transformed into a pentacyclic derivative **39**, which served as a direct precursor of target alkaloids alstilobanines A, E and angustilodine.

**Scheme 14:**
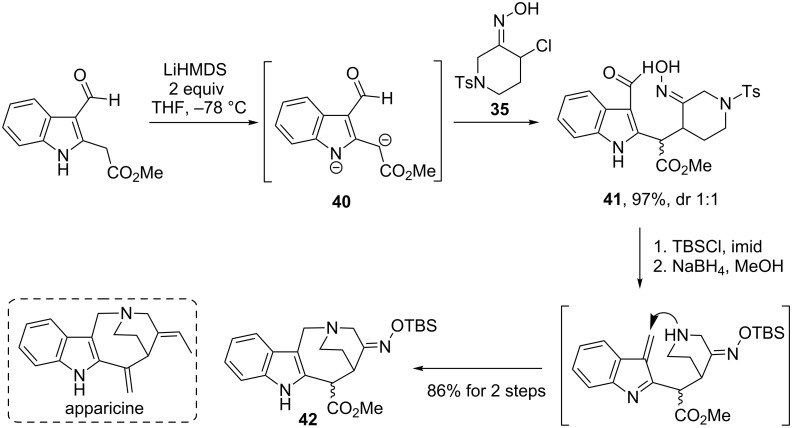
Weinreb’s approach to the core structure of apparicine alkaloids.

The same strategy was employed to construct the tetracyclic core of the apparicine class of indole alkaloids (see Scheme 14) [36]. Here, coupling of indole dianion **40** with chlorooxime **35** (via nitrosoalkene **NSA8)** furnished the corresponding adduct **41** in 97% yield as a separable 1:1 mixture of diastereomers. Each isomer was subjected to cyclization under reductive conditions to form an indol-fused 1-azabicyclo[4.2.2]decane structure **42**.

**Scheme 15:**
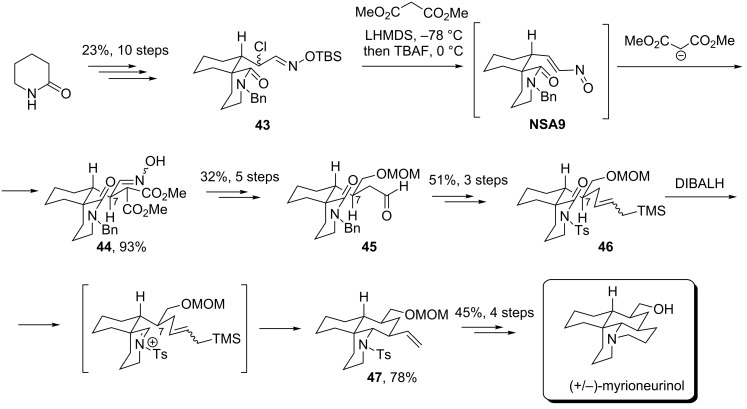
Weinreb’s synthesis of (+/−)-myrioneurinol via stereoselective conjugate addition of malonate to nitrosoalkene **NSA9**.

Conjugate addition of malonate anion to a nitrosoalkene intermediate **NSA9** was used as a key stage to construct an important C(7)–C(8) connection in the total synthesis of (+/−)-myrioneurinol (Scheme 15) [34–35]. α-Chloroxime TBS ether **43** (prepared in 10 steps from δ-valerolactam) reacted with a dimethyl malonate anion under Denmark’s conditions via intermediate **NSA9** to give the corresponding adduct **44** in 93% yield. Importantly, both stereoisomers obtained were oxime *E,Z*-isomers with the C-7 relative configuration being the same as in myrioneurinol. Subsequent deoxygenation and reduction of aldoxime followed by transformation of the malonate unit to the formyl group furnished intermediate **45**, which was subjected to Wittig olefination. Subsequent aza-Sakurai cyclization of intermediate **46** provided tricyclic derivative **47** as a single stereoisomer, which was then transformed into target myrioneurinol by standard operations.

### Addition of organometallic reagents to conjugated nitrosoalkenes

The addition of organometallic compounds to conjugated nitrosoalkenes is a promising strategy towards various α-branched oximes. Unfortunately, these reactions are not sufficiently developed to date and only few reports dealing with a successful Michael addition of organometallic compounds to α-nitrosoalkenes were reported so far.

**Scheme 16:**
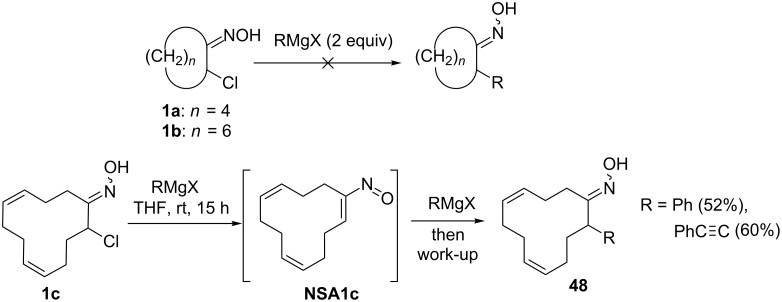
Reactions of cyclic α-chloro oximes with Grignard reagents.

Among the first, Ohno and co-workers studied the addition of Grignard reagents to α-chlorooximes derived from cyclic ketones [23]. In their experiments, 2 equivalents of organomagnesium compound were used with one equivalent needed to transform α-chlorooximes into nitrosoalkene (Scheme 16). Chloro-substituted сyclododeca-4,8-dien-1-one oxime **1c** did produce the desired adducts **48** in moderate yields, yet experiments with 2-chlorocyclohexanone oxime (**1a**) and 2-chlorocyclooctanone oxime (**1b**) were unsuccessful. This early result demonstrates that classical way to generate conjugated nitrosoalkenes from α-chlorooximes may not be compatible with further conjugate addition of Grignard reagents.

**Scheme 17:**
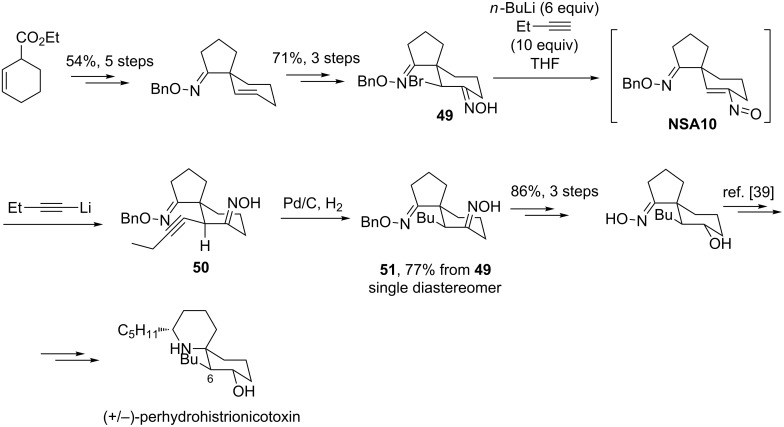
Corey’s synthesis of (+/−)-perhydrohistrionicotoxin.

Despite of these rather disappointing results, in 1977 Corey successfully employed conjugate addition of 1-lithio-1-butyne to a nitrosoalkene intermediate to introduce the butyl group at C-6 atom in the total synthesis of (+/-)-perhydrohistrionicotoxin (Scheme 17) [37–39]. The key precursor of perhydrohistrionicotoxin, dioxime **51**, was prepared by the reaction of α-bromo oxime **49** with 10 equivalents of 1-butyne and 6 equivalents of butyllithium in THF followed by hydrogenation of alkynyl oxime **50**. As a result of these transformations, the desired dioxime **51** is obtained in 77% yield. Important to note, that the addition of a nucleophile to nitrosoalkene **NSA10** occurred exclusively *trans*-stereoselective with respect to *O*-benzyloxy-oxime fragment.

**Scheme 18:**
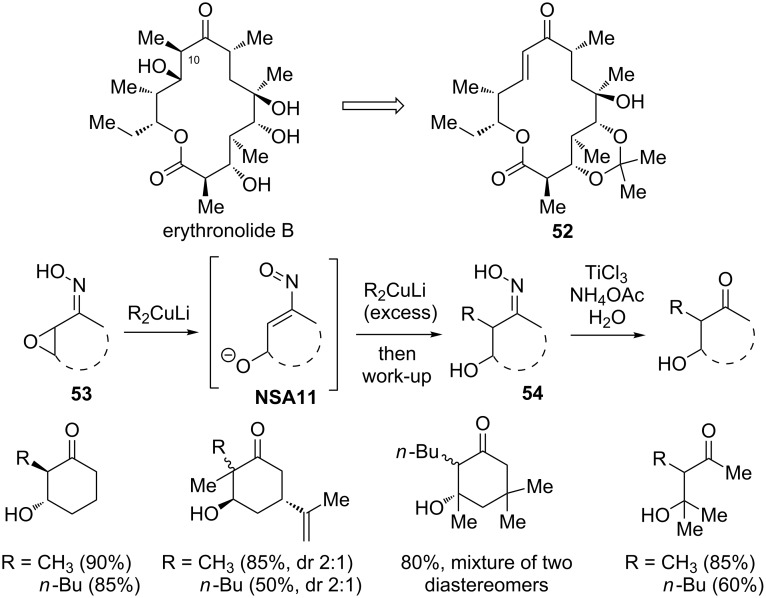
Addition of Gilman’s reagents to α,β-epoxy oximes **53**.

In their studies towards the total synthesis of erythronolide B (an aglycone of erythromycin B) from intermediate **52**, Corey and co-workers suggested the ring opening of α,β-epoxy oximes **53** with Gilman’s reagents as a plausible way to introduce a methyl group at the C-10 position [40] (Scheme 18). Since the addition occurs in α-position and an excess (5 equivalents) of organometallic compound is needed, the reaction is believed to proceed through nitrosoalkene intermediate **NSA11**. The reaction proved to be efficient yielding the desired β-hydroxyoximes **54** in high yields (80–90%). Interestingly, with cyclohexenone oxime excellent stereoselectivity was observed (exclusively *trans* isomers were formed), yet substituted cyclohexenone oxime derivatives such as α,β-epoxycarvone and epoxyisophorone oximes produced diastereomeric mixtures of products. Although, it was not reported whether this strategy was helpful in achieving the synthesis of erythronolide B, this result indicates that the organocopper compounds undergo conjugate addition to nitrosoalkenes in much more selective manner as compared to organolithium and organomagnesium reagents.

**Scheme 19:**
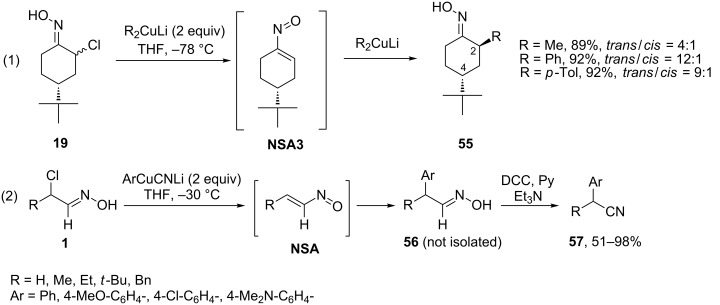
Addition of Gilman’s reagents to α-chlorooximes.

Later on, Weinreb and co-workers demonstrated that organocopper reagents smoothly react with α-chlorooximes [41]. Thus, the reaction of Gilman’s reagent (2 equivalents) with 4-(*tert*-butyl)-2-chlorocyclohexan-1-one oxime (**19**) produced the corresponding α-alkyl- and α-aryl-substituted cyclohexanone oximes **55** in high yields and excellent stereoselectivities (Scheme 19, reaction (1)). After some optimization of the procedure (switching from Gilman’s reagent to aryl lithiocyanocuprates), this method was successfully extended to a wide range of acyclic α-chlorooximes **1** (Scheme 19, reaction (2)). Interestingly, the aryl group, not the cyanide anion is transferred from aryl lithiocyanocuprates to a nitrosoalkene intermediate **NSA**. However, still two equivalents of the organometallic compound were needed to ensure reaction completion. The resulting α-aryl-substituted aldooximes **56** were without isolation converted into the corresponding nitriles **57** by treatment with DCC in the presence of a py/Et_3_N mixture.

**Scheme 20:**
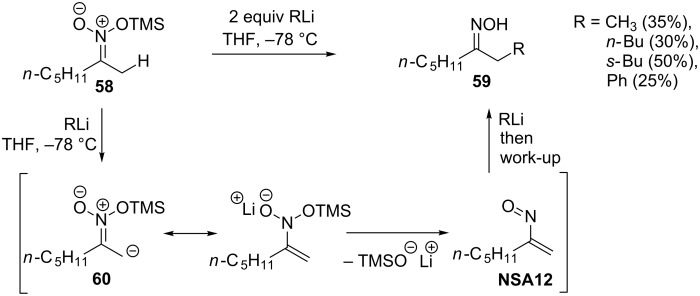
Reaction of silyl nitronate **58** with organolithium reagents via nitrosoalkene **NSA12**.

The use of other nitrosoalkene sources (see Scheme 1) in reaction with organometallic compounds is very limited. Thus, Seebach and co-authors reported the addition of organolithium reagents to O-silyl nitronates **58** leading to α-substituted oximes **59** (Scheme 20) [42]. It is likely, that upon treatment with organolithium reagent silyl nitronate **58** is deprotonated to give anion **60**, which eliminates the silyloxy anion to form nitrosoalkene **NSA12**. Conjugate addition of the second equivalent of the organolithium compound furnishes oximes **59**. However, oximes **59** are produced in rather poor yields.

**Scheme 21:**
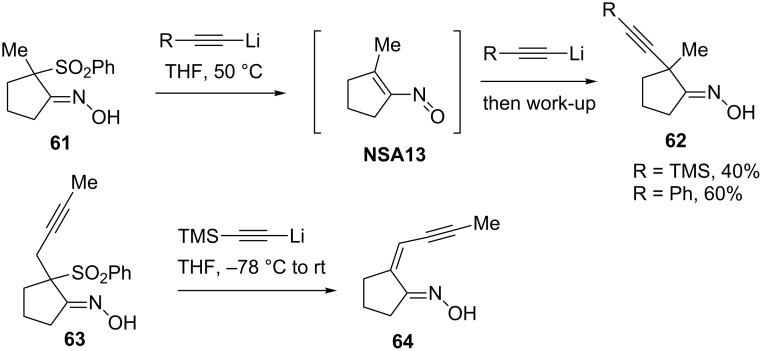
Reaction of β-ketoxime sulfones **61** and **63** with lithium acetylides.

In 1996, Trost reported the use of β-ketoxime sulfones **61** in the reaction with lithium acetylides that resulted in formal substitution of the sulfinate group through an elimination–addition mechanism (Scheme 21) [43]. Interestingly, unlike α-halooximes, 1,4-elimination in sulfones **61** to generate nitrosoalkenes **NSA13** proceeds only at elevated temperatures (50 °C), that may account for relatively low yields of products **62**. Furthermore, the reaction of sulfone **63** with lithium (trimethylsilyl)acetylide furnished only enoxime **64**.

Apparently, the application of more convenient and selective nitrosoalkene sources (e.g., TBS ethers of α-halooximes **2**) in the reaction with organometallic compounds may lead to further developments in this promising methodology.

### Addition of arenes to conjugated nitrosoalkenes

**Scheme 22:**
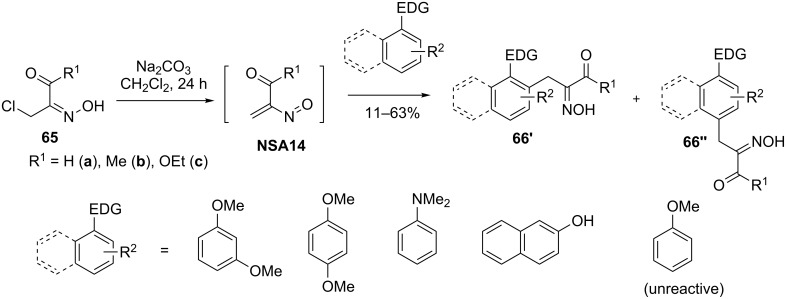
Electrophilic addition of nitrosoalkenes **NSA14** to electron-rich arenes.

Besides common C-nucleophiles, nitrosoalkenes can react with nucleophilic arenes and heteroarenes. Gilchrist and Roberts demonstrated that the reaction of highly electron-rich aromatics with nitrosoalkenes **NSA14** affords the corresponding α-aryl-substituted oximes **66** in low to moderate yields as mixtures of regioisomers [44] (Scheme 22). Nitrosoalkenes were generated from the corresponding α-halooximes **65** upon treatment with Na_2_CO_3_. The efficiency of the addition reaction depends on both electron enrichment of the aromatic ring and the electron-withdrawing character of the substituent in nitrosoalkene **NSA14**. 1,3-Dimethoxybenzene gave the corresponding substitution products in highest yield, while anisole proved to be unreactive under these conditions.

**Scheme 23:**
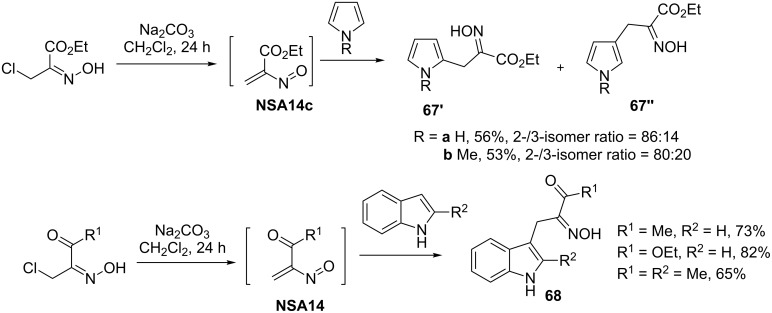
Addition of nitrosoalkenes **NSA14** to pyrroles and indoles.

Electron-rich aromatic heterocyclic compounds such as pyrroles and indoles enter reactions with nitrosoalkenes more smoothly [44–55]). The addition of nitrosoalkenes to pyrroles and indoles is a convenient and mild strategy for the functionalization of these heterocyclic systems. As shown in early reports by Gilchrist [44–45,56], oximinoalkylation of these heterocycles with **NSA14** provides α-hetaryl-substituted oximes **67** and **68** in high yields and with good regioselectivity (Scheme 23). It should be noted, that the formation of α-heteroaryl oximes **67** and **68** can occur not only via an S_E_Ar mechanism, but also through an alternative [4 + 2]-cycloaddition/elimination pathway (for cycloaddition chemistry of nitrosoalkenes see reviews and accounts: [6–10,57–58]).

**Scheme 24:**
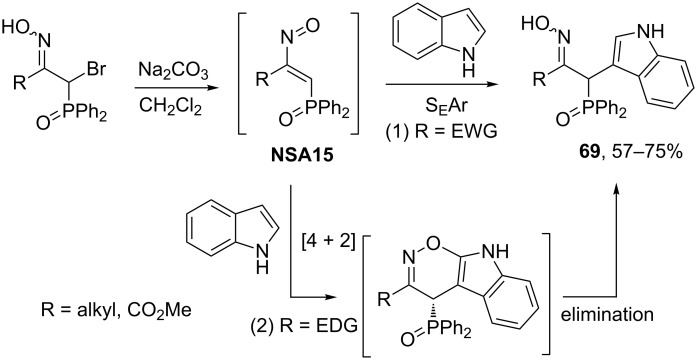
Reaction of phosphinyl nitrosoalkenes **NSA15** with indole.

In the recent work by Palacios and co-workers, the reaction of phosphinyl nitrosoalkenes **NSA15** (derived from the corresponding α-bromooximes) with electron-rich nitrogen heterocycles to give adducts **69** is described and the comparison of electrophilic aromatic substitution (1) and cycloaddition (2) routes is discussed [59] (Scheme 24). Quantum-chemical calculations suggest that the [4 + 2]-cycloaddition process (2) is thermodynamically favored for nitrosoalkenes bearing electron-donating group R, whereas the presence of an electron withdrawing group at the same position promotes the electrophilic substitution pathway (1).

**Scheme 25:**
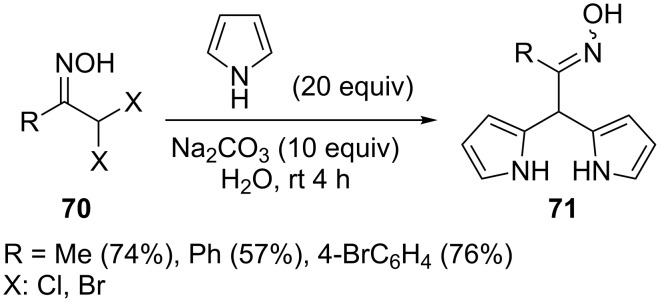
Reaction of pyrrole with α,α’-dihalooximes **70**.

Pinho e Melo and co-workers developed a one-pot double addition of pyrroles (and indoles) to nitrosoalkenes derived from α,α’-dihalooximes **70** [60–62] (Scheme 25). The synthesis of bis-pyrroles **71** is carried out in water as the solvent. As pointed by the authors, heterogenous conditions can accelerate the rate of reaction compared to organic solvent phase.

**Scheme 26:**
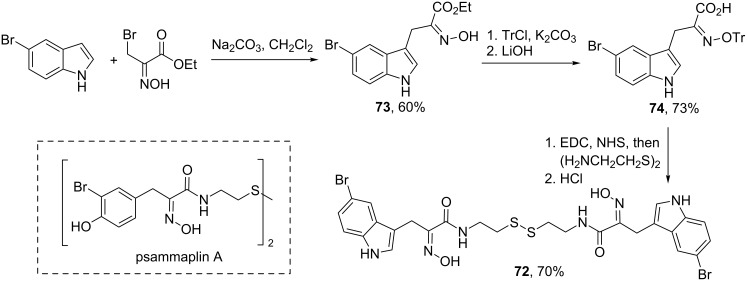
Synthesis of indole-derived psammaplin A analogue **72**.

The described method of oximinoalkylation of the С-3 position of indoles has a great potential for the synthesis of pharmaceutically relevant compounds and natural products. Thus, de Lera and co-workers described the synthesis of indole-derived psammaplin A analogue **72**, which displayed more potent activities than the parent natural product (Scheme 26) [63]. In their synthesis, 5-bromo-1*H*-indole was converted to the corresponding functionalized oxime **73** upon the action of ethyl bromopyruvate oxime in the presence of Na_2_CO_3_ in 60% yield. The adduct was transformed into carboxylic acid **74**, which was then used in a double amidation with cystamine to give the target compound **72** after unmasking of oxime. A series of other psammaplin A analogs were prepared in a similar manner.

**Scheme 27:**
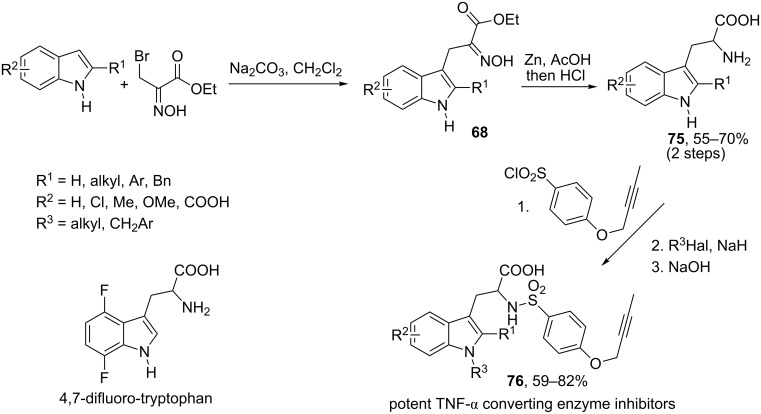
Synthesis of tryptophanes by reduction of oximinoalkylated indoles **68**.

Reduction of the oxime group in oximinoalkylated indoles provides a direct access to various substituted tryptamine derivatives. Thus, reduction of adducts **68** derived from ethyl bromopyruvate oxime and indoles can be considered as a simple and convenient method for the preparation of substituted tryptophanes **75** as shown in Scheme 27 [64].

This synthetic scheme has found several applications in medicinal chemistry. Thus, Park and co-workers successfully employed adducts **68** as intermediates in the synthesis of tumor necrosis factor-α converting enzyme inhibitors **76** [65]. In 2012, Dougherty and co-workers used this approach to prepare 4,7-difluorotryptophan [66].

**Scheme 28:**
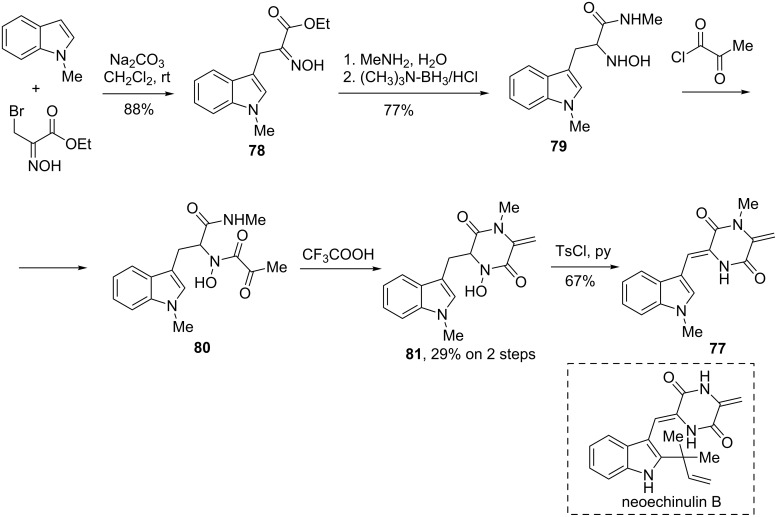
Ottenheijm’s synthesis of neoechinulin B analogue **77**.

A similar strategy was successfully applied by Ottenheijm in the total synthesis of an analogue of the tryptophan-containing natural alkaloid neoechinulin B (indole **77**) [67–68] (Scheme 28). At the initial stage, *N*-methylindole was alkylated with ethyl bromopyruvate oxime and sodium carbonate to give adduct **78**, which was then transformed into *N*-hydroxytryptophan derivative **79** using aminolysis and reduction with (CH_3_)_3_N·BH_3_ complex [47,49,69]. Acylation of **79** with pyruvoyl chloride gave amide **80**, which was converted into dioxopiperazine **81** upon the action of trifluoroacetic acid. Subsequent water elimination furnished the title neoechinulin B analogue **77**.

**Scheme 29:**
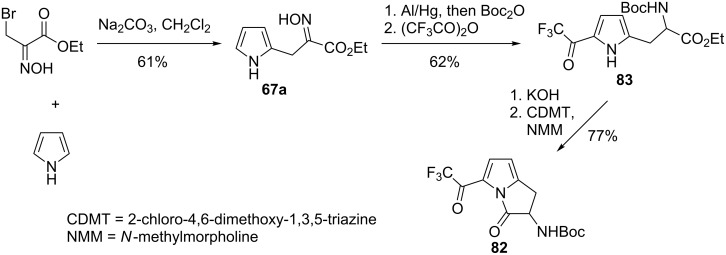
Synthesis of 1,2-dihydropyrrolizinones **82** via addition of pyrrole to ethyl bromopyruvate oxime.

Gilchrist [70] employed oximinoalkylation of pyrrole as the initial stage in the synthesis of 1,2-dihydropyrrolizinone antibiotic **82** [71–73] (Scheme 29). The addition of ethyl 2-nitrosoacrylate (generated from ethyl bromopyruvate oxime) to pyrrole under basic conditions afforded product **67a** in 61% yield. Subsequent reduction/protection of oxime and selective trifluoroacetylation provided 2,5-disubstituted pyrrole **83**, which was cyclized to target dihydropyrrolizin-3-one **82**.

**Scheme 30:**
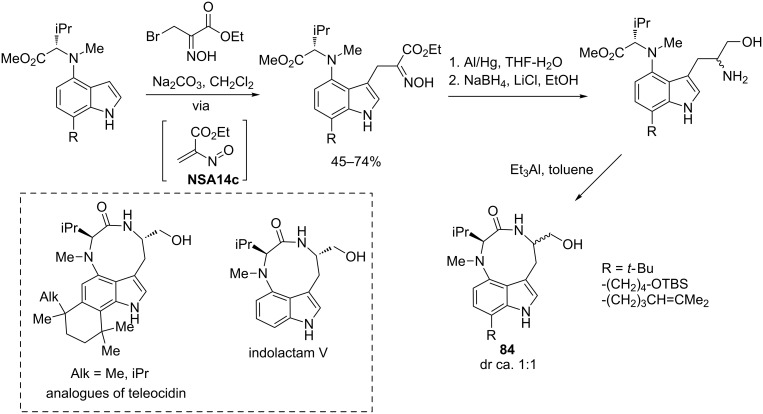
Kozikowski’s strategy to indolactam-based alkaloids via addition of indoles to ethyl bromopyruvate oxime.

Kozikowski [74–75] used the advantage of selective oximinoalkylation of indoles to construct the core structure of indolactam-based alkaloids **84** (activators of protein kinase C) as shown in Scheme 30. Employing the same strategy, Webb [76] and Resnick [77] prepared analogues of teleocidin and later Quick [78] accomplished the synthesis of indolactam V.

### Tandem C-nucleophile addition/cyclization processes involving conjugated nitrosoalkenes

With certain C-nucleophiles possessing additionally an electrophilic center a Michael addition to nitrosoalkenes can be followed by a cyclization. These processes are of synthetic value since bioactive five- and six-membered N–O hetereocycles are formed.

**Scheme 31:**
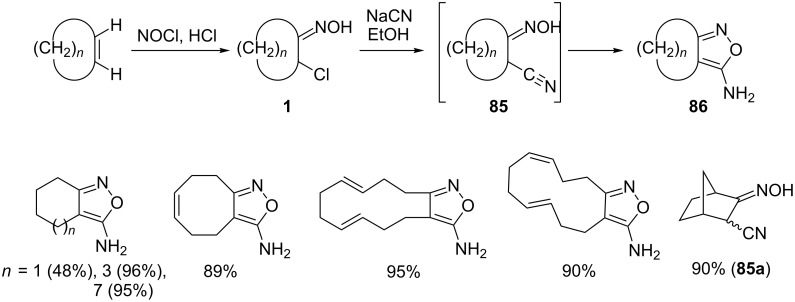
Addition of cyanide anion to nitrosoalkenes and subsequent cyclization to 5-aminoisoxazoles **86**.

A characteristic example of such a process is the Michael addition of cyanide anions to nitrosoalkenes. Ohno and colleagues [79] reported that reaction of **NSA** derived from oximes of cyclic chloroketones **1** with sodium cyanide led to the corresponding fused 5-aminoisoxazoles **86** rather than the expected α-cyanooximes **85** (Scheme 31). It is believed that the initially formed α-cyanooximes **85** undergo rapid cyclization to isoxazole derivatives **86**. Interestingly, in the case of a more sterically hindered 3-chloronorcamphor oxime, the corresponding α-cyanooxime **85a** was obtained, which did not undergo cyclization to isoxazole. Considering the fact that cyclic chloroketones are prepared by chloronitrosylation of cyclic olefins, the overall process is a highly straightforward strategy for the conversion of alkenes to bioactive 5-aminoisoxazoles [80].

**Scheme 32:**
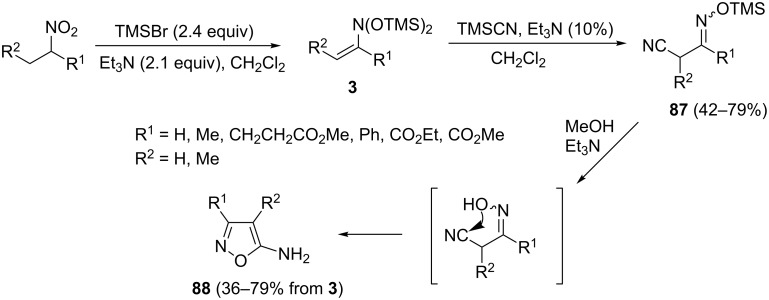
Et_3_N-catalysed addition of trimethylsilyl cyanide to *N*,*N*-bis(silyloxy)enamines **3** leading to 5-aminoisoxazoles **88**.

Our group employed *N*,*N*-bis(silyloxy)enamines **3** as sources of nitrosoalkenes in reaction with trimethylsilylcyanide in the presence of triethylamine as catalyst (Scheme 32) [81]. Interestingly, the initial addition products are intercepted by TMSCN forming stable TMS ethers of α-cyanooximes **87**, which can be isolated by vacuum distillation. Mild desilylation of **87** initiates the intramolecular cyclization to 5-aminoisoxazoles **88**, which were obtained in moderate to good yields. Following this synthetic scheme, 5-aminoisoxazoles **88** can be readily accessed from available nitroalkanes in three steps.

**Scheme 33:**
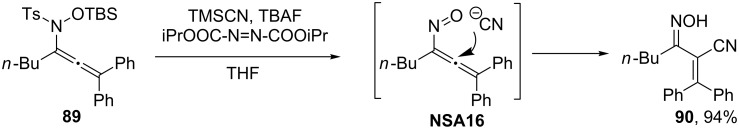
Addition of TMSCN to allenyl *N*-siloxysulfonamide **89**.

Recently, Tanimoto and co-workers [22] reported the addition of TMSCN to a nitrosoallene intermediate **NSA16** generated from allenyl *N*-siloxysulfonamide **89** in the presence of TBAF and diisopropyl azodicarboxylate. Interestingly, the addition product **90** does not undergo cyclization (Scheme 33).

**Scheme 34:**
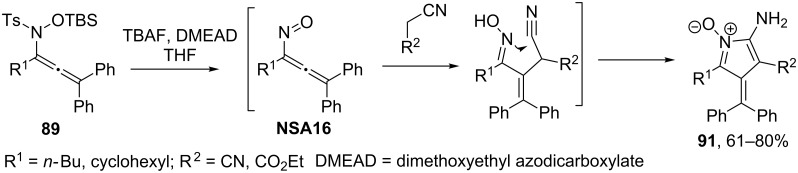
Reaction of nitrosoallenes **NSA16** with malodinitrile and ethyl cyanoacetic ester.

At the same time, reactions of nitrosoallenes of type **NSA16** with malodinitrile and ethyl cyanoacetic ester afforded azafulvene derivatives **91** (Scheme 34) [82]. Remarkably, the formation of five-membered cyclic nitrone through N-attack of the oxime on the cyano group is more preferable over the six-membered N–O heterocycle.

**Scheme 35:**
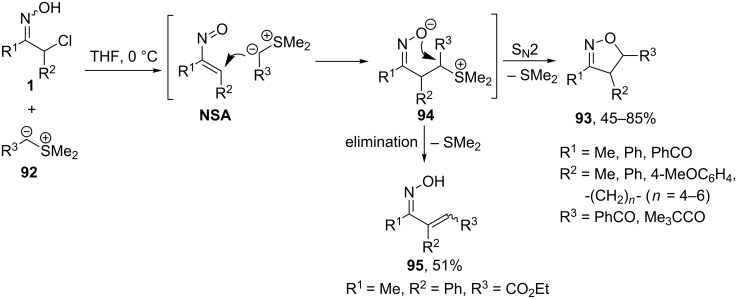
[4 + 1]-Annulation of nitrosoalkenes **NSA** with sulfonium ylides **92**.

C-Nucleophiles possessing leaving groups have been also used in the construction of N–O heterocycles using nitrosoalkenes as intermediates. An early example is the reaction of sulfonium ylides **92** with α-halo ketoximes **1** leading to isoxazolines **93** reported by Bravo [11] and Gilchrist [83] (Scheme 35). The process is believed to proceed via the generation of a nitrosoalkene intermediate (**NSA**) followed by a formal [4 + 1]-annulation reaction with ylide (tandem Michael addition/intramolecular nucleophilic substitution of dimethylsulfide by oximate anion in intermediate **94**).

The addition of sulfonium ylides to nitrosoalkenes can end up not only with cyclic products, but also with α,β-unsaturated oximes **95**, if the elimination of dimethyl sulfide from **94** proceeds faster than the cyclization. The cyclization/elimination selectivity was found be highly dependent on the nature of substituents in ylide **92**. Only when R^3^ was an acyl or phenacyl group, exclusive formation of isoxazoline products **93** was observed.

Triphenylarsonium ylides were also studied in the reaction with nitrosoalkenes, yet lower yields of isoxazolines **93** were obtained as compared to sulfonium ylides [11].

**Scheme 36:**
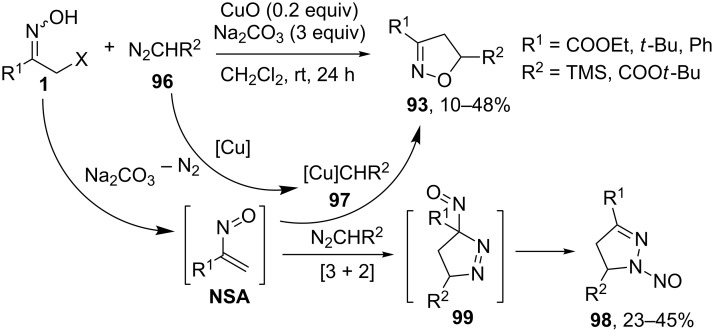
Reaction of diazo compounds **96** with nitrosoalkenes **NSA**.

Following the same reaction pattern, Cheng and co-workers [84] applied diazo compounds **96** instead of sulfonium ylides **92** in the reaction with α-bromo ketoximes **1** (Scheme 36). The reaction requires a copper catalyst, which transforms the diazo compound **96** into a metal carbene complex **97**. The latter reacts with a nitrosoalkene intermediate **NSA** (formed from α-bromo ketoxime **1**) producing isoxazoline **93** with recovery of the catalyst. Unfortunately, the reaction is complicated by a concurrent [3 + 2]-cycloaddition of diazo compounds **96** to nitrosoalkenes leading to *N*-nitrosopyrazoles **98** via intermediates **99**.

**Scheme 37:**
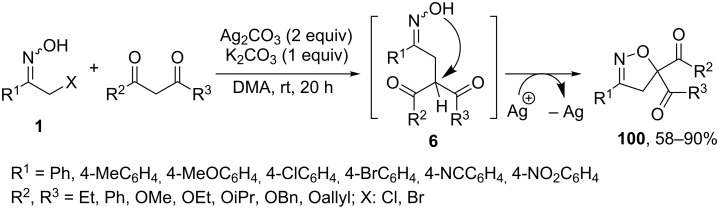
Tandem Michael addition/oxidative cyclization strategy to isoxazolines **100**.

A substantial improvement of this isoxazoline ring-forming strategy was recently introduced by Li et al. [85], who achieved the oxidative [4 + 1]-annulation of nitrosoalkenes with 1,3-dicarbonyl compounds (Scheme 37). Optimized reaction conditions require 2 equivalents of silver carbonate as oxidizer and K_2_CO_3_ as a base to generate nitrosoalkene from a halooxime precursor **1**. The plausible mechanism involves the initial conjugate addition of dicarbonyl compounds to a nitrosoalkene followed by a silver-mediated radical cyclization of the resulting oximes **6** to final isoxazolines **100**. Importantly, the reaction is well-tolerated by various substituents both in halooxime and in dicarbonyl compounds producing isoxazolines **100** in moderate to high yields.

## Conclusion

In conclusion, the Michael addition of carbon-centered nucleophiles to conjugated nitrosoalkenes is a promising strategy for the construction of C–C bonds that has been already demonstrated by its use in numerous total syntheses of natural products and pharmaceutically relevant compounds. However, a number of challenges still remain to be addressed in this methodology, namely: (1) the extension of the scope of C-nucleophiles that can be involved in conjugate addition to nitrosoalkenes; (2) the development of general methods for coupling nitrosoalkenes with organometallic reagents; (3) the elaboration of tandem and domino-processes utilizing conjugate addition of C-nucleophiles to nitrosoalkenes and finally, (4) the design of catalytic enantioselective versions of the Michael addition to nitrosoalkenes (for advances in catalytic asymmetric cycloadditions of nitrosoalkenes see [86–87]). The use of highly selective precursors of nitrosoalkenes such as silyl ethers of halooximes, *N*-vinyl nitrosoacetals and *N*-vinyl *N*-siloxysulfonamides definitely holds the key to solving these problems.
